# Estudio descriptivo y validación de un modelo predictivo de severidad en pacientes con infección por SARS-CoV-2

**DOI:** 10.1515/almed-2021-0006

**Published:** 2021-05-13

**Authors:** Yolanda Villena-Ortiz, Marina Giralt, Laura Castellote-Bellés, Rosa M. Lopez-Martínez, Luisa Martinez-Sanchez, Alba Estela García-Fernández, Roser Ferrer-Costa, Francisco Rodríguez-Frias, Ernesto Casis

**Affiliations:** Departamento de Bioquímica Clínica, Laboratoris Clínics, Hospital Universitari Vall d’Hebron, Barcelona, España

**Keywords:** COVID-19, modelo logístico, SARS-CoV-2

## Abstract

**Objetivos:**

Durante la pandemia causada por el virus SARS-CoV-2 ha surgido la necesidad de identificar variables predictivas que permitan una rápida identificación de aquellos pacientes que desarrollarán la COVID-19 severa para una rápida intervención. Este estudio ha desarrollado y validado un modelo capaz de realizar un pronóstico de severidad de la COVID-19.

**Métodos:**

A partir de datos analíticos, demográficos y comorbilidades de pacientes visitados en el Servicio de Urgencias con sintomatología compatible de COVID-19, se ha realizado un estudio descriptivo y comparativo de pacientes con PCR-RT positiva y negativa para SARS-CoV-2 y de pacientes con enfermedad COVID-19 moderada y severa. La cohorte COVID-19 positiva ha servido para el desarrollo de un modelo de regresión logística.

**Resultados:**

Se han incluido 410 pacientes COVID positivo (303 con enfermedad moderada y 107 con enfermedad severa) y 81 COVID negativo. Las variables predictivas del modelo son: lactato deshidrogenasa, proteína C reactiva, proteínas totales, urea y plaquetas. La calibración interna mostró un área bajo la curva ROC (AUC) de 0,88 (IC95%: 0,85–0,92), con un porcentaje de clasificaciones correctas del 85,2% a un valor de corte de 0,5. La validación externa (100 pacientes) obtuvo un AUC de 0,79 (IC95%: 0,71–0,89), con un 73% de clasificaciones correctas.

**Conclusiones:**

El modelo predictivo desarrollado permite seleccionar desde el Servicio de Urgencias, con una única extracción de sangre y con magnitudes habituales en un Laboratorio Clínico, aquellos pacientes que con mayor probabilidad desarrollarán COVID-19 severa, proporcionando una importante herramienta para la planificación y la toma de decisiones clínicas.

## Introducción

En diciembre de 2019, China informó a la Organización Mundial de la Salud sobre un grupo de casos de neumonía de etiología desconocida en la región de Wuhan (Hubei, China) [1]. Posteriormente, el “Chinese Center for Disease Control” identificó el agente causal como un β coronavirus, al que se denominó internacionalmente como SARS-CoV-2 y el cuadro clínico resultante como COVID-19 [2]. Previamente a la pandemia actual, dos β coronavirus fueron identificados como la causa de brotes epidémicos limitados: SARS-CoV-1 en 2003 y Middle East Respiratory Syndrome coronavirus (MERS-CoV) en 2012, con tasas de mortalidad de alrededor del 10% y del 36%, respectivamente [3]. Los coronavirus son una familia de virus de origen zoonótico que pueden causar diversos cuadros clínicos, desde un resfriado común a síndromes agudos severos respiratorios (SARS) que provocan tanto infecciones pulmonares como manifestaciones extrapulmonares [4].

La mayoría de los pacientes SARS-CoV-2 son asintomáticos o presentan síntomas leves, pero un 20% de los casos desarrollan una enfermedad pulmonar grave, caracterizada por fiebre, tos, disnea, infiltraciones pulmonares y síndrome respiratorio agudo, así como manifestaciones extrapulmonares [5]. En algunos pacientes, la infección está asociada a fenómenos tromboembólicos y a una respuesta exacerbada del sistema inmunitario, provocando un aumento desproporcionado de la liberación de citoquinas pro-inflamatorias, que se ha descrito como el síndrome de “tormenta de citoquinas” [6], [7]. Estas dos situaciones, junto a la insuficiencia respiratoria, se han relacionado con un aumento de los ingresos en la Unidad de Cuidados Intensivos (UCI) y un incremento de la mortalidad [8].

La infección por SARS-CoV-2 se ha convertido en un corto espacio de tiempo en una pandemia de consecuencias impredecibles constituyendo una amenaza para la salud pública [9]. Desde el inicio, ha sido una prioridad la búsqueda e identificación de variables clínicas y de laboratorio predictoras de formas severas de la enfermedad que permitan la intervención inmediata en los casos graves y la optimización de recursos humanos y técnicos. La utilización de estos predictores permite la estratificación del riesgo en una situación donde la capacidad de hospitalización, tanto en unidades de menor como de mayor complejidad, puede estar limitada.

Se han identificado como variables clínicas relacionadas con un peor pronóstico la edad avanzada de los pacientes, la presencia de enfermedad cardiovascular (ECV) o enfermedad pulmonar obstructiva crónica (EPOC), la diabetes mellitus (DM), la hipertensión arterial (HTA), la dislipemia (DLP) y la insuficiencia renal (IR) [10], [11].

El laboratorio clínico ha sido una pieza fundamental en la estratificación de la gravedad y el pronóstico de la enfermedad. Junto a la disminución de la presión y saturación de oxígeno, diversas magnitudes biológicas se han utilizado para este fin. Entre ellas, la disminución del contaje de linfocitos, el aumento de marcadores inflamatorios como ferritina, proteína C reactiva (PCR) e interleucina 6 (IL-6), el aumento de tiempo de protrombina (INR) y dímero D (DD), el aumento de enzimas como lactato deshidrogenasa (LDH), creatina quinasa (CK) y aminotransferasas, entre otros [12].

Esta situación ha propiciado la proliferación de modelos predictivos y de herramientas de inteligencia artificial como ayuda al diagnóstico, seguimiento y pronóstico [13, 14].

Los objetivos de este estudio han sido: 1) Realizar un análisis descriptivo de una cohorte de pacientes con cuadro clínico compatible con infección por SARS-CoV-2 que fueron atendidos en el Servicio de Urgencias, del Hospital Universitari Vall d’Hebron 2) Desarrollar y validar un modelo predictivo que permita identificar a partir de una primera analítica aquellos pacientes SARS-CoV-2 positivos que pueden mostrar mayor gravedad: ingreso en UCI y mortalidad.

## Materiales y métodos

### Diseño del estudio y selección de pacientes

Estudio retrospectivo realizado entre los días 23 y 30 de abril de 2020 con pacientes que acudieron al Servicio de Urgencias del Hospital Universitari Vall d’Hebron con sintomatología compatible de infección por SARS-CoV-2. Se procedió en todos ellos a una extracción sanguínea y un exudado nasofaríngeo y orofaríngeo para la detección del virus SARS-CoV-2 en el momento del ingreso mediante PCR a tiempo real (PCR-RT).

Los pacientes se clasificaron en dos grupos en función del resultado de la PCR-RT: COVID positivo y COVID negativo. Todos los pacientes COVID positivos fueron ingresados en el hospital. Estos últimos se clasificaron en dos subgrupos, moderados y severos, en función de la gravedad de la enfermedad. Se consideró severidad si cumplían criterios clínicos de ingreso en la UCI y/o *exitus*.

Los criterios de exclusión de pacientes fueron: la falta de acceso a la información clínica, la ausencia de alguna magnitud o parámetro de laboratorio incluidos en la Tabla 1 y la positivización de la PCR-RT durante la hospitalización de un paciente clasificado previamente como COVID negativo.

**Tabla 1:** Magnitudes y parámetros de laboratorio incluidos en el estudio y características sobre la medición.

Magnitudes y parámetros	Sistema analítico	Espécimen	Contenedor
Interleucina 6, pg/mL	Cobas 411 (Roche)	Plasma	BD Vacutainer^®^ Barricor con heparina de litio

Hemoglobina, g/dL	Sysmex XN-1000 (Roche)	Sangre total	BD Vacutainer^®^EDTAK2
Hematocrito			
Amplitud de distribución eritrocitaria, %			
Recuento de leucocitos, ×10^9^/L			
Recuento de neutrófilos, ×10^9^/L			
Recuento de linfocitos, ×10^9^/L			
Recuento de monocitos, ×10^9^/L			
Recuento de plaquetas, ×10^9^/L			

Dímero-D, ng/mL	ACLTOP 750 (Werfen)	Plasma	BD Vacutainer^®^ con citrato de sodio
Tiempo de protrombina, INR			
Fibrinógeno derivado, g/L			

Alanina aminotransferasa, UI/L	AU5800 (Beckman Coulter)	Plasma	BD Vacutainer^®^ Barricor con heparina de litio
Aspartato aminotransferasa, UI/L			
Bilirrubina directa, mg/dL			
Bilirrubina total, mg/dL			
Calcio, mg/dL			
Creatinina, mg/dL			
Ferritina, ng/mL			
Glucosa, mg/dL			
Lactato deshidrogenasa, UI/L			
Potasio, mmol/L			
Proteína C reactiva, mg/dL			
Proteínas totales, g/dL			
Sodio, mmol/L			
Urea, mg/dL			

Coronavirus SARS-CoV-2	Reacción en cadena de la polimerasa (PCR-RT). CFX96^TM^ Real-Time System (BioRad)	Exudado nasofaríngeo y orofaríngeo	Contenedor con medio de transporte específico

Los datos demográficos, antecedentes patológicos y comorbilidades fueron obtenidos del sistema de gestión de datos del hospital. Las comorbilidades consideradas fueron: HTA, DLP, DM, IR, EPOC y obesidad.

Este estudio ha sido aprobado por el Comité de Ética del Hospital Universitari Vall d’Hebron.

### Magnitudes y parámetros de laboratorio

Las magnitudes estudiadas, el sistema analítico empleado y el espécimen utilizado se muestran en la Tabla 1.

### Análisis estadístico

#### Estudio descriptivo

Se realizó un estudio descriptivo de los diferentes grupos comparando, por un lado, los pacientes COVID positivos con los pacientes COVID negativos y, por otro, los pacientes COVID positivos moderados con los pacientes COVID positivos severos. Las variables comparadas fueron edad, sexo, comorbilidades y las magnitudes analíticas incluidas en la Tabla 1.

Las variables cuantitativas se expresaron como la mediana y el rango intercuartil (IQR). En la comparación se utilizó el test de Mann**–**Whitney para las variables continuas y el test de Chi cuadrado para las variables dicotómicas.

#### Modelo predictivo

El análisis de regresión logística multivariante para predecir severidad se realizó a partir del grupo COVID positivo (moderados y severos), siguiendo las recomendaciones de la guía “Transparent Reporting of a multivariable prediction model for Individual Prognosis Or Diagnosis” (TRIPOD) [14]. En el modelo se incluyeron, en primer lugar, todas las variables con capacidad predictiva en el análisis univariante y que a nuestro criterio y en concordancia con la literatura predicen severidad y peor pronóstico [15]. El número máximo de variables predictoras que se incluyeron en el modelo multivariante se definió con el criterio de Peduzzi [16] calculando los eventos por variable (EPV) como el número de eventos entre el número de variables independientes, siendo este cociente superior o igual a 10. Se ejecutó un método de selección de variables por pasos mixto con metodología atrás/adelante.

El diagnóstico del modelo se realizó incluyendo el estudio de independencia de los errores (inspección visual de los residuales calculados frente a los valores predichos), la ausencia de multicolinealidad entre variables predictoras mediante el índice de factor de inflación de la varianza (VIF), el control de los valores alejados y los valores influyentes (análisis de los residuales estudentizados, valores de influencia “*leverage*” y los índices de influencia ∆Beta [17], ∆*χ* ^2^ y ∆Dev [18]), la interacción entre variables predictoras (evaluando todos los posibles términos de interacción a partir de un modelo máximo inicial jerárquico y eliminando las interacciones si el resultado de la prueba era superior a 0,05) y la evaluación de la linealidad de las variables con el logit (inspección visual mediante categorización de los predictores cuantitativos según *ntiles* y asignación a cada categoría del valor de la mediana).

La calibración del modelo se realizó mediante la prueba de Hosmer y Lemeshow. Se desglosaron las probabilidades predichas en 5 grupos asumiendo que el modelo ajustaba si p>0,10. Se estableció como requisito de aplicación que la mayoría de las frecuencias esperadas en cada grupo fuesen superiores a 5 y ninguna de ellas inferior a 1.

La validación interna del modelo se llevó a cabo mediante dos procedimientos. En primer lugar, se aplicó un método de validación cruzada basado en la división aleatoria de los datos en k grupos (n=5), estimando el modelo y calculando la medida de bondad de ajuste para dichos grupos. En segundo lugar, se calculó del área bajo la curva *Receiver Operating Characteristic* (AUC) por remuestreo *bootstrap* [14].

El análisis descriptivo y de regresión fue realizado utilizando el programa estadístico Stata (versión Stata/IC 15).

### Validación externa del modelo predictivo

La validación externa (VE) del modelo se realizó con un grupo adicional de 100 pacientes COVID positivos que acudieron entre los meses de junio y octubre de 2020 al Servicio de Urgencias del Hospital Universitari Vall d’Hebron, ingresados en el hospital y que cumplían los criterios de inclusión aplicados en la realización del modelo.

Se aplicó el método predictivo obtenido al grupo de validación, se calculó el AUC y se estimó la pérdida de predicción respecto al modelo original (pérdida de predicción = AUC – AUC_VE_). El modelo se consideró fiable si la pérdida de predicción es inferior al 10%.

## Resultados

De los 517 pacientes seleccionados, 20 fueron descartados por cumplir alguno de los criterios de exclusión, 410 (82,5%) fueron positivos y 87 (17,5%) fueron negativos por PCR-RT para SARS-CoV-2. Entre los pacientes positivos, 303 (73,9%) desarrollaron enfermedad moderada y 107 (26,1%), severa. En este último grupo, 61 pacientes (57,0%) fueron *exitus* durante el estudio.

Las características demográficas, las comorbilidades asociadas y la comparación de estas variables entre el grupo con enfermedad moderada y el grupo con enfermedad severa se muestran en la Tabla 2. La mediana de edad de los pacientes COVID positivos fue de 61 años (IQR: 48–74), el 55,4% eran hombres y las comorbilidades asociadas más frecuentes fueron: HTA (41,2%), DLP (27,3%), DM (18,8%) y cardiopatía (10,7%). La mediana de edad fue, respectivamente, de 69 (55–78) y de 60 años (48–73) en los pacientes con enfermedad severa y moderada (p<0,05). En el grupo con enfermedad severa, se observó una mayor proporción de hombres (66,4%) y mayor frecuencia de todas las comorbilidades asociadas, a excepción de la patología pulmonar previa. Este aumento fue estadísticamente significativo en la IR y en la cardiopatía (p<0,05).

**Tabla 2:** Características demográficas y comorbilidades de los pacientes positivos para SARS-CoV-2: pacientes con enfermedad moderada y severa.

		Total (n=407)	Moderado (n=303)	Severo (n=107)	p-valor
**Edad**		61,0 (48,0–74,0)	60,0 (48,0–73,0)	69,0 (55,0–78,0)	0,0001

**Sexo**	Mujer	183 (44,6%)	147 (48,5%)	36 (33,6%)	0,008

**Comorbilidades**	Hipertensión arterial	169 (41,2%)	117 (38,6%)	52 (48,6%)	0,071
	Dislipemia	112 (27,3%)	77 (25,4%)	35 (32,7%)	0,145
	Diabetes mellitus	77 (18,8%)	55 (18,2%)	22 (20,6%)	0,853
	Insuficiencia renal	20 (4,9%)	11 (3,6%)	9 (8,4%)	0,048
	Cardiopatía	44 (10,7%)	25 (8,3%)	19 (17,8%)	0,006
	Enfermedad pulmonar	32 (7,8%)	27 (8,9%)	5 (4,7%)	0,160
	Obesidad	31 (7,6%)	20 (6,6%)	11 (10,3%)	0,216
	Otras	19 (4,6%)	12 (4,0%)	7 (6,5%)	0,275

En las variables dicotómicas los resultados se muestran como el número de pacientes y porcentajes y la comparación entre grupos como el p-valor del test Chi cuadrado. La edad se expresa como la mediana y rango intercuartil y la comparación entre grupos como el p-valor del test de Mann**–**Whitney.

La comparación de los valores obtenidos de las diferentes magnitudes y parámetros analíticos entre los pacientes COVID negativos y COVID positivos se muestra en la Tabla 3. En la Tabla 4, se presenta la misma comparación entre los pacientes con enfermedad moderada y severa. En el grupo de pacientes COVID positivos la mediana de los valores fue significativamente más elevada para el fibrinógeno, alanina aminotransferasa (ALT), aspartato aminotransferasa (AST), bilirrubina directa, bilirrubina total, ferritina, glucosa, LDH, PCR, IL-6 y más baja en la amplitud de distribución eritrocitaria (ADE), recuento de leucocitos, recuento de linfocitos, calcio, potasio y sodio.

**Tabla 3:** Comparación de magnitudes y parámetros analíticos entre pacientes COVID positivo y COVID negativo.

Magnitudes y parámetros	Pacientes COVID− (n=81)	Pacientes COVID+ (n=410)	p-valor	Mann**–**Whitney
Interleucina 6, pg/mL	16,2 (7,8–36,9)	49,7 (27,0–83,8)	0,000	−6,771
Hemoglobina, g/dL	12,8 (11,4–14,4)	13,6 (12,4–14,7)	0,004	−2,919
Hematocrito	40,4 (35,3–43,5)	41,5 (38,4–44,6)	0,015	−2,444
Amplitud de distribución eritrocitaria, %	13,2 (12,6–15,4)	13,1 (12,5–13,9)	0,048	1,98
Recuento de leucocitos, ×10^9^/L	7,7 (6,0–9,9)	6,7 (5,2–8,8)	0,003	2,973
Recuento de neutrófilos, ×10^9^/L	5,2 (4,1–7,6)	5,0 (3,6–7,0)	0,251	1,148
Recuento de linfocitos, ×10^9^/L	1,4 (1,0–2,1)	1,0 (0,8–1,4)	0,000	4,91
Recuento de monocitos, ×10^9^/L	0,6 (0,4–0,9)	0,4 (0,3–0,6)	0,000	6,05
Recuento de plaquetas, ×10^9^/L	243 (185–307)	210 (165–262)	0,002	3,076

Dímero-D, ng/mL	244 (149–582)	281,5 (183,0–543,5)	0,108	−1,607
Tiempo de protrombina, INR	1,05 (0,99–1,12)	1,10 (1,03–1,16)	0,000	−3,652
Fibrinógeno derivado, g/L	4,7 (4,1–5,4)	5,65 (4,82–6,39)	0,000	−5,129

Alanina aminotransferasa, UI/L	20 (15–28)	30,0 (19,0–49,8)	0,000	−4,216
Aspartato aminotransferasa, UI/L	26 (20–33)	41 (30–57)	0,000	−7,208
Bilirrubina directa, mg/dL	0,27 (0,22–0,33)	0,32 (0,25–0,39)	0,000	−3,923
Bilirrubina total, mg/dL	0,54 (0,44–0,76)	0,62 (0,48–0,78)	0,075	−1,778
Calcio, mg/dL	9,2 (9,0–9,6)	9,0 (8,7–9,3)	0,000	4,209
Creatinina, mg/dL	0,80 (0,62–0,99)	0,82 (0,66–0,96)	0,281	−1,078
Ferritina, ng/mL	179 (94–519)	589,0 (300,5–1088,3)	0,000	−6,272
Glucosa, mg/dL	100 (89–113)	108 (96–130)	0,003	−3,007
Lactato deshidrogenasa, UI/L	257 (217–303)	338,5 (281,3–437,0)	0,000	−7,168
Potasio, mmol/L	4,02 (3,67–4,23)	3,83 (3,60–4,05)	0,002	3,181
Proteína C reactiva, mg/dL	2,58 (0,67–8,91)	10,86 (5,05–18,16)	0,000	−6,695
Proteínas totales, g/dL	7,5 (7,1–7,9)	7,6 (7,1–8,0)	0,325	−0,985
Sodio, mmol/L	137,3 (135,4–138,5)	136,2 (134,2–138,2)	0,017	2,395
Urea, mg/dL	32 (24–50)	33,5 (26,0–46,8)	0,786	−0,271

Los valores se expresan como la mediana y el rango intercuartil. La comparación se ha realizado mediante el test de Mann**–**Whitney del cual se muestra el p-valor y el estadístico.

**Tabla 4:** Comparación de magnitudes y parámetros analíticos entre los pacientes COVID positivo moderado y severo.

Magnitudes y parámetros	Moderado (n=81)	Severo (n=410)	p-valor	Mann**–**Whitney
Interleucina 6, pg/mL	42,3 (21,1–65,6)	88,1 (61,2–133,0)	0,000	−8,787
Hemoglobina, g/dL	13,6 (12,6–14,7)	13,4 (12,2–14,6)	0,246	1,161
Hematocrito	41,7 (38,7–44,6)	41,4 (38,2–44,2)	0,365	0,907
Amplitud de distribución eritrocitaria, %	13 (12,4–13,7)	13,4 (13,0–14,4)	0,000	−4,499
Recuento de leucocitos, ×10^9^/L	6,5 (5,1–8,3)	7,4 (5,7–10,9)	0,000	−3,522
Recuento de neutrófilos, ×10^9^/L	4,8 (3,4–6,6)	6,1 (4,6–9)	0,000	−4,902
Recuento de linfocitos, ×10^9^/L	1,1 (0,8–1,5)	0,8 (0,6–1,1)	0,000	5,864
Recuento de monocitos, ×10^9^/L	0,5 (0,3–0,6)	0,4 (0,3–0,5)	0,002	3,081
Recuento de plaquetas, ×10^9^/L	216 (172–267)	195 (145–246)	0,012	2,521

Dímero-D, ng/mL	258,0 (172,5–456,0	424,0 (257,5–739,5)	0,000	−4,72
Tiempo de protrombina, INR	1,1 (1,0–1,2)	1,1 (1,0–1,2)	0,719	−0,36
Fibrinógeno derivado, g/L	5,6 (4,8–6,3)	6,0 (5,0–6,6)	0,019	−2,34

Alanina aminotransferasa, UI/L	29 (19–49)	31 (20–54)	0,3199	−0,995
Aspartato aminotransferasa, UI/L	38 (29–52)	52 (36,5–71)	0,000	−4,991
Bilirrubina directa, mg/dL	0,31 (0,25–0,38)	0,35 (0,27–0,45)	0,0005	−3,505
Bilirrubina total, mg/dL	0,61 (0,48–0,75)	0,64 (0,48–0,86)	0,081	−1,743
Calcio, mg/dL	9,1 (8,8–9,3)	8,8 (8,5–9,0)	0,000	5,331
Creatinina, mg/dL	0,78 (0,64–0,92)	0,96 (0,83–1,30)	0,000	−6,886
Ferritina, ng/mL	481 (260–838)	986 (517–1460)	0,000	−5,158
Glucosa, mg/dL	105 (95–124)	120 (102–145)	0,000	−3,956
Lactato deshidrogenasa, UI/L	314 (268–382)	450 (365–584)	0,000	−8,956
Potasio, mmol/L	3,80 (3,55–4,00)	3,96 (3,66–4,21)	0,001	−3,293
Proteína C reactiva, mg/dL	8,87 (3,83–15,30)	17,86 (11,19–23,57)	0,000	−7,491
Proteínas totales, g/dL	7,7 (7,3–8,0)	7,3 (6,9–7,7)	0,000	5,073
Sodio, mmol/L	136,2 (134,3–138,1)	136,2 (134,2–138,3)	0,687	−0,402
Urea, mg/dL	31,0 (24,0–41,0)	48,0 (33,0–72,8)	0,000	−7,294

Los valores se expresan como la mediana y el rango intercuartil. La comparación se ha realizado mediante el test de Mann**–**Whitney del cual se muestra el p-valor y el estadístico.

Entre los pacientes con enfermedad severa se encontraron valores significativamente más elevados en IL-6, ADE, recuento de leucocitos, DD, fibrinógeno, AST, bilirrubina directa, creatinina, ferritina, glucosa, LDH, potasio, PCR, urea y más bajos en el recuento de linfocitos y plaquetas, calcio y proteínas totales.

**Figura 1:**
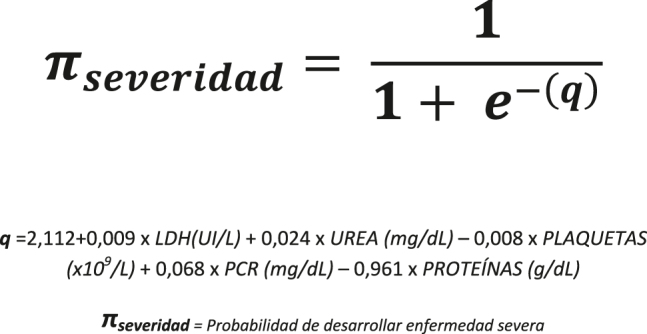
Ecuación del modelo de regresión logística obtenido para la predicción de severidad en pacientes con COVID-19.

En el análisis multivariante, las variables que presentaron en conjunto mayor nivel de significación fueron: LDH, PCR, proteínas totales, urea y recuento de plaquetas. El grado de significación (prueba de Wald) [19] para cada una de ellas fue de 6,43; 3,78; −3,78; 3,83 y −3,48, respectivamente. Con estas variables se cumplía el criterio de Peduzzi, siendo 107 el número de eventos y 5 las variables predictoras, con el objetivo de que el modelo fuese además, lo más parsimonioso posible. La ecuación del modelo logístico [20] se representa en la Figura 1.

La capacidad predictiva del modelo fue de 0,377. La prueba de significación global por el método de máxima verosimilitud indicó que el modelo predice la severidad por la COVID-19 de forma estadísticamente significativa (*χ* ^2^=173,55; *df*=5; p<0,05). En la evaluación de la bondad de ajuste del modelo se obtuvieron los siguientes índices pseudo-*R* ^2^: Cox y Snell [21] de 0,353; Nagelkerke [22] de 0,515 y Mcfadden [23] corregido de 0,351.

**Figura 2:**
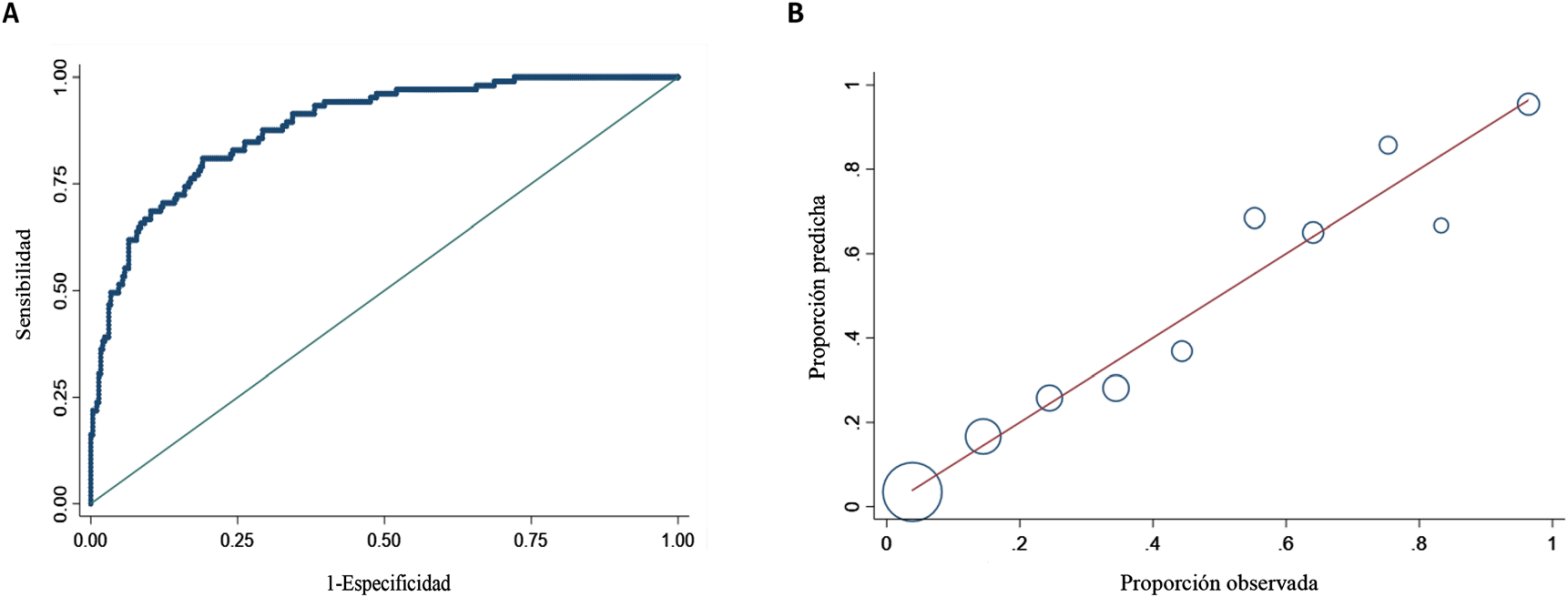
(A) Área bajo la curva ROC, de la capacidad predictiva del modelo para el pronóstico de severidad en pacientes con COVID-19. (B) Test de Hosmer y Lemeshow de calibración del modelo de regresión logística. En el eje de abscisas se muestra la probabilidad de severidad observada y en el eje de ordenadas la probabilidad esperada según lo predicho por el modelo. El tamaño de las burbujas es proporcional al número de pacientes.

Se obtuvo un AUC de 0,885 (IC95%: 0,849–0,921) (Figura 2A). Para un punto de corte de 0,5, la sensibilidad (S) y la especificidad diagnóstica (E), el valor predictivo positivo (VPP) y el valor predictivo negativo (VPN) fueron de 61,9, 93,5, 77,4 y 87,3%, respectivamente. El porcentaje de clasificaciones correctas fue del 85,2%. La S, E, VPP y VPN para un punto de corte de 0,3, fueron de 77,4, 81,6, 60,0 y 90,9%, respectivamente. En este último caso, la S aumenta a expensas del porcentaje de clasificaciones correctas.

En el diagnóstico del modelo, la independencia de los errores se confirmó visualmente (datos no mostrados). La ausencia de multicolinealidad entre variables predictoras quedó confirmada en todas las variables (Â_VIF_=1,2). El control de valores alejados y valores influyentes dio lugar a la exclusión de 2 pacientes del modelo (N_final_=408). La interacción entre variables fue descartada, partiendo de un modelo máximo inicial jerárquico. La evaluación de la linealidad de las variables con el logit fue confirmada visualmente (datos no mostrados).

La prueba de Hosmer y Lemeshow mostró una p=0,521, valor superior al límite que se emplea en ciencias de la salud, por lo que confirmamos que el modelo fue exitosamente calibrado (Figura 2B).

La validación interna fue superada mediante los dos métodos empleados: la validación cruzada obtuvo valores pseudo-R^2^ entre 0,167 y 0,483; y el cálculo del AUC mediante remuestreo *bootstrap* fue de 0,840.

La muestra empleada para la validación externa obtuvo un AUC igual a 0,794 (IC95%: 0,708–0,880), siendo la pérdida de predicción del 9,11%. La S, E, VPP y VPN para un punto de corte de 0,5, fue de 73,7, 72,1, 77,8 y 67,4% respectivamente. El porcentaje de pacientes correctamente clasificados fue del 73,0%. Para un punto de corte de 0,3, la S, E, VPP y VPN fue de 91,2, 51,2, 71,2 y 81,5%. La S y el VPN aumentan a expensas de la E y el VPP. El porcentaje de pacientes correctamente clasificados fue en este caso del 74,0%.

## Discusión

Este estudio presenta datos demográficos, de comorbilidad y analíticos de pacientes con enfermedad COVID-19 que requirieron ingreso hospitalario durante la primera oleada de la pandemia comparando pacientes con pronóstico moderado con aquellos que experimentaron peor evolución. Además, crea un modelo predictivo de evolución desfavorable utilizando parámetros analíticos obtenidos en la primera extracción sanguínea al ingreso en el Servicio de Urgencias.

El riesgo de severidad de la enfermedad incrementa con la edad y el sexo masculino, hallazgos confirmados en este estudio. Según un estudio de mortalidad realizado en diferentes países, incluyendo España [24], la ratio de mortalidad hombre/mujer es de 1,3. Las hipótesis basadas en factores de riesgo (consumo de alcohol, hábito tabáquico, estilo de vida, toma de fármacos, etc.) y comorbilidades que varían según el sexo y la edad podrían ser las explicaciones más plausibles de las diferencias observadas.

Los pacientes con comorbilidades preexistentes tienen más riesgo de tener complicaciones. Las comorbilidades más representadas en este estudio fueron la HTA, la DLP, la DM y las enfermedades cardíacas. En menor proporción, la enfermedad pulmonar, la obesidad y la IR. Sin embargo, solo la existencia de cardiopatía o de IR tuvieron mayor significación para la aparición de formas severas de la enfermedad, ambas situaciones asociadas a un aumento de la mortalidad en estos pacientes [25]. La prevalencia de todas las comorbilidades estudiadas en relación a la enfermedad está ampliamente referenciada en la literatura [26]. Es importante remarcar que la existencia de un estado proinflamatorio y una alteración de la inmunidad innata en estas enfermedades crónicas podría influir en la sintomatología, evolución y pronóstico de la enfermedad [27].

La alteración de diferentes magnitudes biológicas (Tablas 3 y 4) refleja la infección multiorgánica por SARS-CoV-2 debida a la amplia expresión en el organismo de la diana celular del virus, el receptor de la enzima convertidora de angiotensina 2 (ACE2) [28].

El modelo predictivo se construyó con las cinco magnitudes que en conjunto permiten discriminar con mayor significación entre enfermedad severa y moderada: aumento de LDH, PCR y urea y disminución de plaquetas y proteínas totales. Durante el tiempo de estudio, el Servicio de Microbiología de nuestro hospital informó el resultado de la PCR-RT para SARS-CoV-2 únicamente de forma cualitativa, por lo que no disponemos de este dato. Además, no está clara la asociación entre severidad y la carga viral de la muestra tomada del paciente.

La LDH es una enzima ubicua y su aumento refleja destrucción celular y tisular. La concentración aumentada de LDH en plasma se considera un biomarcador de actividad y severidad en la fibrosis pulmonar, siendo marcador pronóstico de enfermedad intersticial severa. En pacientes críticos con COVID-19, el aumento de LDH indica un incremento de la actividad y de la extensión del daño pulmonar [29] y es un marcador de severidad de la enfermedad [30].

La PCR es un reactante de fase aguda y está significativamente incrementada en las fases iniciales de la infección debido a mediadores inflamatorios como la IL-6 y se ha utilizado como variable pronóstica en el síndrome del distrés respiratorio agudo [31]. El incremento de PCR refleja también el proceso de vasculitis sistémica que sufren los pacientes con peor pronóstico. La utilización de este marcador en el pronóstico de los pacientes con COVID-19 ha sido reportada en diferentes estudios de comparación entre pacientes con enfermedad severa y moderada [32], [33] y se ha asociado con un aumento de la mortalidad [34].

La urea y la creatinina son marcadores de función renal. Ambas son filtradas en los glomérulos renales aunque la creatinina apenas se reabsorbe en los túbulos. Es por ello que la urea tiene un papel fisiológico importante en el equilibrio hídrico glomerular y es un marcador más sensible que la creatinina en el diagnóstico de la IR aguda. El incremento de urea plasmática se ha asociado a efectos adversos y mortalidad en pacientes con fallo cardíaco [35], tromboembolismo pulmonar [36], pancreatitis necrotizante [37], hemorragias gastrointestinales [38] y neumonía [39]. Un estudio reciente muestra que valores elevados de urea en el momento de admisión están fuertemente asociados con eventos adversos y mortalidad en enfermos ingresados en UCI, incluso después de la corrección por IR [40]. El aumento de urea en los pacientes con COVID-19 es una variable independiente de pronóstico desfavorable [41], [42], probablemente asociada a IR aguda, causada por una hipoxemia consecuencia del distrés respiratorio o a la acción directa del virus sobre los túbulos renales [43].

La disminución de la concentración de proteínas se interpreta como un marcador subrogado de la concentración plasmática de albúmina y ha sido asociada a mortalidad en pancreatitis, infección, trauma, quemaduras y disfunción hepática. Los mecanismos fisiopatológicos que dan lugar a esta disminución pueden ser secundarios al incremento de la permeabilidad vascular, del volumen de distribución y de la expresión del factor de crecimiento endotelial vascular (VEGF) y a la disminución de la síntesis proteica y del tiempo de vida media de la albúmina. En infecciones agudas, la inflamación incrementa la permeabilidad capilar debido al aumento de citoquinas y a la sobreexpresión del VEGF conduciendo a la expansión del espacio intersticial y al incremento en la distribución de volumen de albúmina [44]. Diversos estudios en pacientes con infección por SARS-CoV-2, muestran que la disminución de la concentración plasmática de albúmina es un factor independiente que predice resultados adversos y mortalidad [45], [46]. No obstante, estos estudios no son capaces de explicar esta condición en la primera analítica en pacientes con COVID-19.

La trombocitopenia ha sido reportada como factor asociado al aumento de mortalidad en pacientes ingresado en UCI [47]. En la infección por SARS-CoV-2 es frecuente la aparición de plaquetopenia en pacientes con enfermedades inflamatorias y metabólicas sistémicas previas. Las causas de esta plaquetopenia no están claras, aunque se apuntan varias hipótesis: 1) activación y agregación de plaquetas debido a un daño pulmonar directo, resultando en la formación de microtrombos y el consumo de plaquetas; 2) inhibición de la síntesis de plaquetas como consecuencia del daño en las células hematopoyéticas de la médula ósea a causa de la inflamación y del propio virus; 3) destrucción plaquetar por el sistema inmune [48]. Un reciente metaanálisis muestra que la plaquetopenia es más prevalente en pacientes con COVID-19 severa y en pacientes que fallecen por esta enfermedad [49]. Queda por demostrar si la plaquetopenia es un factor de riesgo independiente de severidad y muerte en estos pacientes o si es secundaria a fallo multiorgánico. En nuestro estudio, la plaquetopenia estaba presente en los pacientes con mal pronóstico en el momento de ingreso hospitalario. Similares resultados se han obtenido en otro estudio, donde se muestra que la disminución de plaquetas en pacientes severos fue anterior a la aparición de los síntomas [50].

Aunque la capacidad predictiva del modelo es limitada (37,7%), el AUC es elevado (0,885), el porcentaje de clasificaciones correctas es del 85,2% y se obtiene un VPP del 77,4% y un VPN del 87,3%, lo cual representa una buena predicción. También ha sido exitosamente calibrado, siendo los resultados predichos acordes con los reales (Figura 2B).

La validación tanto interna como externa se ha superado correctamente. Sin embargo, en la validación externa ha habido una pérdida de predicción del 9,1%. Esto puede deberse a un tamaño insuficiente de muestra para la validación o a la diferente proporción de pacientes severos en la cohorte original (26,3%) respecto a la de validación (57,0%).

Los modelos predictivos tienen un enorme interés y pueden aportar perspectivas cruciales para los servicios asistenciales y para los responsables de políticas sanitarias. Estos modelos se basan en situaciones y datos subyacentes que pueden cambiar a medida que se actualizan y se revisan los datos. Por lo tanto, tienen un cierto riesgo de sesgo si no se cumplen las condiciones iniciales. En este sentido, es importante conocer los puntos fuertes y las limitaciones de estos modelos.

Este modelo presenta diversas limitaciones. No es aplicable a pacientes con sintomatología leve que no requieren ingreso ya que el estudio se realizó con pacientes que acudieron al hospital en situación crítica durante la primera ola de la pandemia. Otra limitación es que no se ha analizado la coexistencia de varias comorbilidades en un mismo paciente, lo que podría dar lugar a cambios en los resultados expuestos. Tampoco se detallan hábitos no saludables como consumo de alcohol o hábito tabáquico, ni otras comorbilidades (hepatopatías, inmunodeficiencias, neoplasias, etc.) que podrían influir en el curso de la enfermedad. Además no se ha considerado el tiempo transcurrido entre la aparición de los síntomas y la toma de muestra en Urgencias, ya que no se disponía de esta información en la mayoría de los pacientes consultados. Magnitudes como la procalcitonina y la troponina que se asocian con severidad no estaban en el perfil analítico de Urgencias de nuestro hospital para COVID-19, por lo que no han podido ser incluidas en este trabajo. Es posible que la inclusión de dichas magnitudes pudiera mejorar el modelo. Se requieren estudios posteriores que contemplen esa posibilidad.

A pesar de las limitaciones, el modelo permite seleccionar desde el Servicio de Urgencias con solamente una extracción sanguínea y con magnitudes analíticas habituales en un Laboratorio Clínico aquellos pacientes que con alta probabilidad desarrollarán un cuadro severo de la enfermedad.
